# A perspective on intermittent fasting and cardiovascular risk in the era of obesity pharmacotherapy

**DOI:** 10.3389/fnut.2025.1524125

**Published:** 2025-01-17

**Authors:** Aristides G. Eliopoulos, Kalliopi K. Gkouskou, Konstantinos Tsioufis, Despina Sanoudou

**Affiliations:** ^1^Department of Biology, Medical School, National and Kapodistrian University of Athens, Athens, Greece; ^2^Center for New Biotechnologies and Precision Medicine, Medical School, National and Kapodistrian University of Athens, Athens, Greece; ^3^Genosophy S.A., National and Kapodistrian University of Athens Spin-off Company, Athens, Greece; ^4^1st Department of Cardiology, Hippokration Hospital of Athens, National and Kapodistrian University of Athens, Athens, Greece; ^5^Clinical Genomics and Pharmacogenomics Unit, 4th Department of Internal Medicine, Medical School, National and Kapodistrian University of Athens, Athens, Greece; ^6^Biomedical Research Foundation of the Academy of Athens, Athens, Greece

**Keywords:** intermittent fasting, precision medicine, anti-obesity pharmacotherapy, cardiovascular disease, genetics, GLP-1, GIP

## Abstract

Intermittent fasting has been linked to metabolic health by improving lipid profiles, reducing body weight, and increasing insulin sensitivity. However, several randomized clinical trials have shown that intermittent fasting is not more effective than standard daily caloric restriction for short-term weight loss or cardiometabolic improvements in patients with obesity. Observational studies also suggest cardiovascular benefits from extended rather than reduced eating windows, and indicate that long-term intermittent fasting regimens may increase the risk of cardiovascular disease mortality. In this perspective, we discuss evidence that may support potential adverse effects of intermittent fasting on cardiovascular health through the loss of lean mass, circadian misalignment and poor dietary choices associated with reward-based eating. Given the ongoing revolution in obesity pharmacotherapy, we argue that future research should integrate anti-obesity medications with dietary strategies that confer robust benefits to cardiometabolic health, combine exercise regimens, and consider genetic factors to personalize obesity treatment. Comprehensive approaches combining diet, pharmacotherapy, and lifestyle modifications will become crucial for managing obesity and minimizing long-term cardiovascular risk.

## 1 Introduction

Intermittent fasting (IF) has gained attention as an effective strategy for reducing body weight and improving metabolic health. IF includes various protocols, such as time-restricted eating (TRE), alternate-day fasting (ADF), and the 5:2 diet (1). TRE confines food intake to a specific window each day, typically less than 12 h, and involves fasting for the remaining hours. ADF alternates between days of normal eating and days with significant calorie restriction or complete fasting. The 5:2 diet involves eating *ad libitum* for 5 days and restricting caloric intake for two nonconsecutive days each week. Several lines of clinical, epidemiological and experimental evidence have suggested that short-term application of IF protocols may improve risk factors associated with cardiovascular disease (2–5). These benefits have been attributed to weight loss, improved lipid profiles, reduced oxidative stress and systolic blood pressure that have been extensively reviewed elsewhere (3, 6, 7).

However, several randomized clinical trials (RCTs) (8–11) and a recent meta-analysis of RCTs (12) have provided robust evidence that neither TRE nor ADF or 5:2 IF is more effective than standard daily caloric restriction in terms of short-term reductions in body weight or improvements in cardiometabolic risk factors in patients with obesity. Likewise, a 3-week ADF regimen in lean adults was reported to be less effective at reducing body fat and improving cardiometabolic health parameters than an equivalent daily energy restriction (13). These observations suggest that many of the short term health-promoting effects of IF are most likely mediated by caloric restriction. Indeed, an 8-week ADF intervention study in overweight patients failed to improve cardiometabolic risk factors in the absence of caloric restriction (14).

In this perspective, we critically evaluate the effects of IF on cardiovascular health, focusing on potential adverse outcomes that may be associated with loss of lean mass, circadian misalignment, and dietary patterns driven by reward-based eating. These links are examined also in the context of the transformative advances in obesity pharmacotherapy, by discussing how pharmacological agents could be combined with dietary interventions for the management of cardiometabolic risk. By synthesizing current evidence, we aim to provide a comprehensive understanding of the role of IF in cardiovascular health and stimulate discussions about optimized personalized dietary strategies for obesity management in the era of obesity pharmacotherapy.

## 2 Emerging concerns of intermittent fasting for cardiovascular health

The scarcity of observational studies addressing the effects of long-term application of IF on cardiovascular health has been recognized as a major gap in nutrition research (1). Intriguingly, a recently published observational analysis of data from the National Health and Nutrition Examination Survey (NHANES) revealed that an eating window extending beyond 11 h is associated with lower cardiovascular disease (CVD) mortality in adults with heart failure (15). Along the same lines, an independent preliminary analysis of data from the NHANES presented at the 2024 American Heart Association conference by Zhong et al. (16) suggested that adhering to an 8-h TRE schedule is associated with a 91% increased risk of CVD mortality among a cohort of over 20,000 adults. The mortality risk was found to be particularly pronounced in individuals with preexisting heart conditions (16). Despite potential limitations, such as confounding factors and reliance on self-reported data which may introduce errors, these studies raise concerns about the long-term impact of IF on cardiovascular health.

Experimental findings in rodents align with the conclusions of these observational studies. Thus, rats subjected to ADF for a prolonged period of time (i.e., 6 months, equivalent to approximately 14 years in humans) developed reduced left ventricular diastolic compliance, a 3-fold increase in interstitial myocardial fibrosis and diminished cardiac reserve (17). Together, these findings underscore the need to better appreciate the behavioral and biological processes linking IF to cardiovascular health and disease.

## 3 Putative behavioral and biological processes that may link intermittent fasting to cardiovascular disease

**FIGURE 1:**
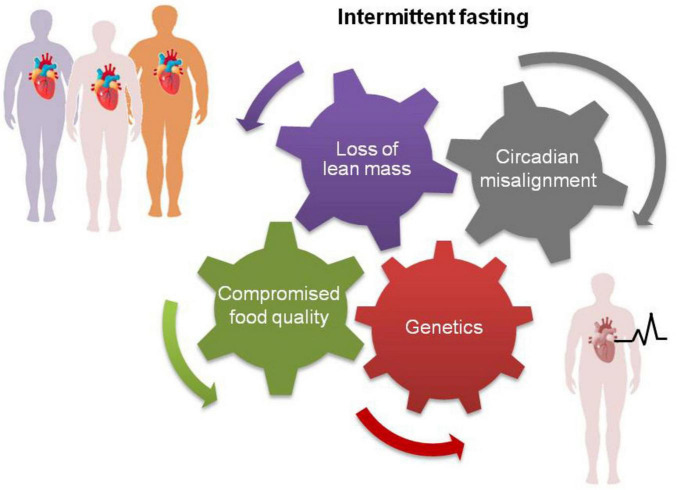
Some people may experience adverse cardiovascular effects following long-term application of intermittent fasting (bottom right) because of higher loss of lean mass, circadian misalignment of food consumption and/or compromised food quality linked to reward eating. These factors, separately or in combination, may particularly impact individuals with preexisting heart conditions or with genetic susceptibility to CVD leading to higher risk of mortality (15, 16).

We suggest that increased loss of lean mass, circadian misalignment of food consumption and/or compromised food quality may underpin the adverse effects of IF on cardiovascular health in genetically predisposed individuals (Figure 1).

### 3.1 Compromised food quality associated with reward-based eating

An RCT in metabolically healthy patients with obesity reported that the limited eating window of IF may lead to increased food consumption on fast days (10). This behavior, termed “reward-based eating,” may degrade the overall diet quality by including more satisfying rather than nutritionally balanced options (18). Indeed, some studies have reported reduced cognitive restraint, reduced fiber intake and greater sugar and meat consumption in individuals on IF regimes than in those on continuous caloric restriction (19–22). We speculate that at least some of the instances of compromised food quality could be attributed to reward eating. Poor food choices may also explain the elevated levels of LDL-cholesterol, a risk factor for CVD, reported in obesity patients undergoing a 12-month ADF but not in patients receiving a continuous hypocaloric diet (10).

Protein consumption tends to also increase among individuals practicing IF (23–25), potentially to increase satiety. While plant-based protein sources are known to benefit cardiovascular health, increased intake of animal protein has been linked to CVD and all-cause mortality (26, 27). Exaggerated consumption of animal-based foods such as red meat, eggs, and dairy is expected to increase choline and carnitine intake, leading to the production of trimethylamine-N-oxide (TMAO) through a metabolic pathway involving the gut microbiota and the liver. Elevated levels of circulating TMAO are associated with a higher incidence of atherosclerotic CVD and major adverse cardiovascular events (28, 29). Although a definitive link between prolonged IF and TMAO has not been established, elevated TMAO levels have been reported in patients with obesity undergoing 5:2 IF for 4 weeks (30).

### 3.2 Loss of lean mass

Loss of lean mass is another factor that may link IF to adverse cardiovascular outcomes. An RCT in patients with overweight or obesity found that a 12-week hypocaloric TRE regimen led to a greater loss of lean mass compared to a conventional hypocaloric diet (9). The reduction in lean mass accounted for approximately 65% of total weight loss (9) which is notably higher than the typical 20–30% range observed with standard hypocaloric diets (31), and primarily involved loss of skeletal muscle mass. Reduced muscle mass has been associated with an increased risk of CVD (32, 33), as well as cardiovascular events and mortality (34). These findings highlight the need for caution when considering IF for individuals at risk of sarcopenia, such as the elderly and cancer patients.

### 3.3 Chrononutrition

Circadian misalignment in food intake may also impact metabolic and cardiac health (35), suggesting additional putative links between IF and CVD. Several observational and interventional studies have shown that eating breakfast is overall associated with cardiometabolic health (36), whereas skipping breakfast increases the risk of CVD (37, 38) and type 2 diabetes (T2D) (39). The behavior of eating breakfast is highly heritable, with twin studies indicating a 56% heritability rate (40). In a Mendelian randomization analysis of data from 200,000 individuals, genetically predisposed breakfast skipping was causally linked to obesity (41). Circadian misalignment caused by irregular eating patterns, such as skipping breakfast during IF, may exacerbate metabolic dysfunction in genetically predisposed individuals. For example, the polymorphism 3111T/C in circadian-related gene *CLOCK* has been associated with differences in glucose tolerance and lipid metabolism (42), both of which are critical to cardiovascular risk. These findings highlight the potential risks of skipping breakfast, particularly for those with a genetic predisposition, and underscores the importance of considering genetic factors when assessing the health impacts of IF and related dietary practices.

Similar to breakfast skipping, late-night eating has been linked to arterial stiffness (43) and to a greater risk of CHD in a 16-year cohort study (38). A recent observational analysis from the NutriNet-Santé study, which included over 103,000 adults, found that for each hour breakfast was delayed after 8:00 a.m. or the last meal was consumed after 8:00 p.m., the overall risk of CVD increased, regardless of body weight (44). This study also revealed a potential protective association between a longer night-time fasting duration and cardiovascular health, only when it was coupled with early first and last meals but not with skipping breakfast (44). Different TRE protocols have assigned varying eating time windows (e.g., early 8 am–2 pm or 12 pm–8 pm), and the multitude of differences in study designs and participant selection render them largely incomparable (45). In the real world, unsupervised individuals’ self-selection of the eating time window is the standard practice. Since the health impacts of different durations of exposure to IF diets can be important, future studies need to investigate this parameter extensively.

Chrononutrition is an important modulator of peripheral clocks in the circadian system controlling blood pressure rhythms (46), partly through the hypothalamus–pituitary–adrenal (HPA) axis. Cortisol is the main hormone produced by HPA. A small-scale intervention study indicated that while morning cortisol is reduced in females who skip breakfast as part of their intermittent diet regimen, postprandial cortisol levels later in the day are higher than those in females who do not skip breakfast (47). The long-term impacts of such shifts in diurnal cortisol remain undefined, but elevated cortisol levels are linked to hypertension and glucose intolerance, which are known risk factors for CVD (48). Indeed, cortisol activates the rennin/angiotensin/aldosterone system (RAAS), a complex hormonal network influencing blood pressure, fluid and electrolyte balance, and systemic vascular resistance. The activation of the RAAS, particularly through angiotensin II, can influence myocardial energy metabolism. For individuals with heart failure, especially those with compromised left ventricular function, the vasoconstrictive effect of angiotensin II may be detrimental by increasing blood pressure, impairing cardiac output and exacerbating heart failure symptoms (49). Therefore, the effects of skipping different meals in the framework of TRE (e.g., dinner *versus* breakfast) require further investigations through controlled studies to elucidate how meal timing impacts HPA axis rhythmicity and cardiovascular risk factors, including fluctuations in blood pressure and glucose levels. Notably, the application of an early (8 a.m. to 5 p.m.) TRE schedule for 1 week lowers fasting glucose, whereas late feeding (12 p.m. to 9 p.m.) has no effect (50).

One of the well-established effects of cortisol is the reduction of brain-derived neurotrophic factor (BDNF), which physiologically functions to inhibit appetite and food intake (51). Compared to continuous caloric restriction, cognitive restraint is reduced in individuals following IF (21) but the effects of intermittent diets on basal BDNF levels remain inconclusive (52). Importantly, larger cortisol-to-BDNF ratios have been associated with cardiometabolic risk and silent ischemia in African Americans (53, 54). *BDNF* is highly polymorphic, and the most common genetic variation, rs6265 (Val66Met), has been associated with greater severity of coronary artery disease (55). Experimental studies in homozygous Val66Met knock-in mice have confirmed this association and demonstrated that alterations in basal cardiac gene expression profiles are associated with the development of diastolic dysfunction (56). It would be interesting to examine whether carriers of BDNF rs6265 in NHANES who have incorporated TRE into their lifestyle are at higher risk of death from CVD compared with noncarriers (15, 16).

### 3.4 Clinical implications and research gaps

The aforementioned mechanisms underscore the importance of cautious implementation of IF strategies because of their potential links with cardiovascular risk. In a clinical setting, the loss of lean mass associated with some IF protocols, could exacerbate sarcopenia in older adults or individuals with chronic conditions and compromise cardiovascular resilience. Similarly, deregulated chrononutrition caused by irregular eating patterns, including genetically-predisposed breakfast skipping or late-night eating, should be taken into consideration, as it is strongly associated with metabolic dysfunction, elevated blood pressure, and increased cardiovascular risk. Reward-based eating should also be monitored among individuals practicing IF, as it may lead to poor dietary patterns that contribute to atherosclerosis and adverse cardiometabolic outcomes.

To address these concerns, future research should prioritize large-scale, longitudinal studies evaluating the long-term cardiovascular impacts of IF, particularly in vulnerable populations such as the elderly or those with preexisting cardiovascular conditions. Further exploration into the comparative effects of different IF protocols, such as TRE and ADF, on eating behavior, metabolic health, and circadian alignment is essential to delineate the conditions under which IF may pose risks or confer benefits.

## 4 Intermittent fasting in the era of obesity pharmacotherapy

From a therapeutic perspective, the controversy surrounding the health outcomes of IF should also be considered in the context of the evolving development of novel anti-obesity and anti-diabetic medications. These drugs include (but are not limited to) the glucagon-like peptide-1 (GLP-1) receptor agonists liraglutide and semaglutide; the dual GLP-1 and GIP (glucose-dependent insulinotropic polypeptide) receptor agonist tirzepatide; and the tri-agonist retatrutide, which activates GLP-1, GIP and glucagon receptors. Their remarkable efficacy in reducing both body weight and obesity-related comorbidities, including CVD and overall mortality (57–60), places IF-based interventions in a new context.

Indeed, pharmacotherapy with GLP-1 receptor or dual GIP/GLP-1 receptor agonists in patients with obesity achieves impressive reductions in body weight in the range of 15–25% compared with TRE or other nutritional interventions, which are typically in the range of 3–4% (1, 61). For example, in a 72-week clinical trial (61), a weekly dose of 15 mg of tirzepatide led to 21% total body weight loss compared with 3% in the placebo group receiving only a hypocaloric diet. Obesity pharmacotherapy reduces food intake and enhances satiety without the need to alter meal timing (62, 63), thus avoiding the potential health risks and reward-based eating behaviors reportedly associated with IF (44, 45). Several studies have also demonstrated that, unlike TRE which decreases lean mass, treatment with tirzepatide, liraglutide or semaglutide reduces body weight with small effects on lean mass (61, 64, 65). Therefore, the available evidence suggests that obesity pharmacotherapy may mitigate some of the behavioral and biological processes observed with IF that could elevate CVD risk.

Indeed, although long-term monitoring of the adverse effects of anti-obesity medications is still needed, data from clinical trials supports favorable efficacy *versus* safety profiles and demonstrates significant improvements in several cardiometabolic health parameters (57–61). Overweight or obesity patients without T2D but with preexisting CVD experience a marked decrease in the incidence of cardiovascular death, nonfatal myocardial infarction, or nonfatal stroke while on semaglutide treatment (66, 67). Moreover, obesity pharmacotherapy significantly reduces cardiovascular events in patients with type 2 diabetes (T2D) (68, 69).

A mixed strategy incorporating both pharmacotherapy and dietary interventions may offer the most comprehensive and pragmatic approach to obesity and cardiometabolic risk management. Along these lines, the SURMOUNT-3 phase III clinical trial revealed that patients with obesity or overweight who achieved ≥5.0% weight reduction with intensive lifestyle intervention prior to treatment with tirzepatide benefited the most from pharmacotherapy (70). Optimizing long-term dietary interventions will also be required to support and maintain the metabolic and cardiovascular benefits achieved during treatment. Incorporating genetics into this optimization process could provide a more precise and personalized approach, ensuring maximum effectiveness and sustainability of critically required lifestyle changes (71–73).

## 5 Discussion and future directions

The prevailing view that IF is cardioprotective largely stems from experimental rodent models and intervention studies (2–4). However, it is important to note that rodents have a significantly greater basal metabolic rate; thus, the reported beneficial metabolic effects of time-restricted eating on blood pressure and cardiac structure in mice and rats (4) may not fully translate to humans. Intermittent fasting studies performed in animals also lack the behavioral aspects of food choice and intake that typify humans, including reward-based eating. Moreover, it is widely recognized that TRE intervention studies in patients with obesity have focused primarily on short-term effects (i.e., durations of less than 12 weeks) and have small sample sizes (1), leaving a gap in our understanding of the safety and effectiveness of long-term intermittent caloric restriction. The observational analyses conducted by Zhong et al. (16) and Billingsley et al. (15) underscore the need for further investigations into the long-term impacts of intermittent diets on cardiovascular health in additional large-scale cohorts.

A major goal for future research will be to identify dietary interventions that both optimally support patients with obesity during pharmacotherapy and maintain the metabolic and cardiovascular benefits achieved during treatment. Given that the health effects and long-term consequences of IF remain unclear (1, 16), we propose that hypocaloric Mediterranean diets may serve as the preferred starting point. Mediterranean diets have been strongly associated with both body weight control and improved long-term cardiovascular outcomes, including clinically meaningful reductions in coronary heart disease, ischemic stroke, and overall CVD mortality (74, 75). Among several popular diets evaluated in a recent meta-analysis (76), the Mediterranean diet had the most consistent and robust evidence of a beneficial effect on both anthropometric parameters and cardiometabolic risk factors.

In addition to Mediterranean dietary interventions, the integration of exercise and behavioral modifications in new pharmacotherapy protocols needs to be considered for their potential to enhance both the achievement and maintenance of reduced body weight. Increased physical activity in combination with reduced caloric intake enables additional weight loss, better glycemic control, and improved insulin sensitivity and lipid profiles (77, 78). Emerging evidence suggests that adherence to physical activity recommendations while on liraglutide leads to a significant, albeit limited, reduction in body weight (79). Future clinical trials should thus aim to identify the optimal combination of diet, physical activity protocols, and behavioral modification techniques to be combined with new pharmacotherapies, as this combination could have a multiplier beneficial effect on the management of obesity.

In conclusion, we herein underscore the need for caution in applying intermittent fasting as a long-term dietary strategy for cardiovascular health. While short-term benefits of IF, such as weight loss and improvements in lipid profiles, have been demonstrated, these effects appear primarily mediated by caloric restriction rather than unique attributes of intermittent fasting. Importantly, the long-term impacts of IF remain largely unexplored. Emerging observational evidence indicates cardiovascular benefits from extended rather than reduced eating windows and increased mortality risk associated with long-term application of TRE. Such adverse effects may be linked to the loss of lean mass, circadian misalignment and poor dietary choices associated with reward-based eating in genetically-predisposed individuals (Figure 1). Conversely, advances in obesity pharmacotherapy offer highly effective alternatives for managing body weight and cardiometabolic risk, yet their long-term safety profiles also require better evaluation.

As we continue to navigate the intricate balance between diet, pharmacotherapy, and cardiovascular risk, it is essential that future research prioritizes the development of integrative approaches that not only address the immediate challenges of obesity but also mitigate long-term cardiometabolic risks, ensuring that treatment strategies are both effective and sustainable. We propose that such a balanced strategy should incorporate evidence-based dietary patterns with proven cardiometabolic benefits, such as Mediterranean diets. Underpinned by genetic and nutritional counseling, the integration of genetic parameters influencing the response to dietary components, type of exercise, different pharmacotherapies and behavioral traits, such as adherence and reward eating, as well as their interactions, will enable further personalization and greater potential for improved pharmacotherapy outcomes in patients with obesity (73, 80–83).

## Data Availability

The original contributions presented in this study are included in this article/supplementary material, further inquiries can be directed to the corresponding author.
